# Strongly Iridescent Hybrid Photonic Sensors Based on Self-Assembled Nanoparticles for Hazardous Solvent Detection

**DOI:** 10.3390/nano8030169

**Published:** 2018-03-16

**Authors:** Ayaka Sato, Yuya Ikeda, Koichi Yamaguchi, Varun Vohra

**Affiliations:** Department of Engineering Science, the University of Electro-communications, 1-5-1 Chofugaoka, Chofu, Tokyo 182-8585, Japan; s1413079@edu.cc.uec.ac.jp (A.S.); b1366569@crystal.ee.uec.ac.jp (Y.I.); kyama@ee.uec.ac.jp (K.Y.)

**Keywords:** hazardous organic solvents, photonic nanostructures, self-assembly, polymer nanoparticles, biomimetic solvent sensors, iridescence

## Abstract

Facile detection and the identification of hazardous organic solvents are essential for ensuring global safety and avoiding harm to the environment caused by industrial wastes. Here, we present a simple method for the fabrication of silver-coated monodisperse polystyrene nanoparticle photonic structures that are embedded into a polydimethylsiloxane (PDMS) matrix. These hybrid materials exhibit a strong green iridescence with a reflectance peak at 550 nm that originates from the close-packed arrangement of the nanoparticles. This reflectance peak measured under Wulff-Bragg conditions displays a 20 to 50 nm red shift when the photonic sensors are exposed to five commonly employed and highly hazardous organic solvents. These red-shifts correlate well with PDMS swelling ratios using the various solvents, which suggests that the observable color variations result from an increase in the photonic crystal lattice parameter with a similar mechanism to the color modulation of the chameleon skin. Dynamic reflectance measurements enable the possibility of clearly identifying each of the tested solvents. Furthermore, as small amounts of hazardous solvents such as tetrahydrofuran can be detected even when mixed with water, the nanostructured solvent sensors we introduce here could have a major impact on global safety measures as innovative photonic technology for easily visualizing and identifying the presence of contaminants in water.

## 1. Introduction

Organic solvents are widely used in multiple industries and for daily chores. For instance, they are employed for industrial printing, coatings, adhesives, painting or cleaning, and in numerous types of advanced technological research and production such as the organic electronics field [1,2,3,4]. A large number of these organic solvents are considered as hazardous and can lead to either environmental or health issues [1,2,3,4,5,6,7,8]. Chlorinated solvents such as chloroform (CF) or chlorobenzene (CB), as well as heterocyclic ethers such as tetrahydrofuran (THF), can damage numerous organs including liver, kidneys, and the central nervous system. Although strict rules on hazardous solvent waste disposal are in place in developed countries, this may not be the case on a global scale. Consequently, developing simple and low-cost technologies to visually detect the presence of contaminants (hazardous organic solvents and their vapors) in air or in water is of major importance for reducing the risks that result from organic solvent usage.

Photonic crystals found in nature have well-defined nano- or micro-structures [9,10,11,12,13,14,15] and have inspired strategies for the development of biomimetic sensing technologies based on structural coloration [13,14,15,16,17]. In particular, some animals provide perfect examples of visible color-tunable photonic materials [10,11,12]. The panther chameleons (*Furcifer Pardalis*) can, for instance, change their skin color by modifying the lattice parameters of their iridophores between the excited and the relaxed states [10]. Various methods for fabricating bio-inspired materials with similar structural coloration properties have been explored over the past two decades, which are either based on lithographic techniques (e.g., electron-beam lithography, imprint lithography, holographic lithography, or two-photon lithography) [18,19,20,21] or on self-assembled materials [22,23,24]. Although lithographic techniques can generate well-defined architectures, colloidal self-assembly has been drawing increasing interest in the field, as it provides a low-cost alternative to fabricating materials with structural coloration properties. This was first achieved with silicon-based monodisperse spherical particles with submicrometer diameters [25,26], but these hard particles were quickly replaced with soft polymer nanoparticles, as they can be produced at a lower cost and their properties can be easily adapted through simple chemistry (e.g., to generate core-shell structures with different refractive indices) [16,23,27].

Several studies have demonstrated that polymer nanoparticles as fused films or embedded in elastomer matrices can be applied to water or organic solvent sensors fabrication [16,27,28]. Similarly to the chameleon’s camouflage properties, the color variation observed from these films can be correlated with changes in the lattice parameters of the photonic crystals and/or changes in refractive indices upon diffusion of the organic molecules inside the matrix. The spectral changes are generally described by the Bragg diffraction equation that is derived from Bragg and Snell’s laws (Equation (1)), which relates the maximum peak wavelength (*λ*) with the incident angle (*θ*) and the effective refractive index (*n*_eff_):

(1) $$m\lambda =2d_{111}\sqrt{n_{\mathrm{eff}}{}^{2}-\sin^{2}\theta }$$

in which *d*_111_ corresponds to the distance between adjacent nanoparticle centers in the (111) plane and *m* is the order of diffraction. *d*_111_ and *n*_eff_ can be calculated following Equations (2) and (3).

(2) $$d_{111}=\sqrt{2/3}D_{\mathrm{particle}}$$

(3) $$n_{\mathrm{eff}}{}^{2}=\sqrt{0.74\;n_{\mathrm{particle}}^{2}+0.26\;n_{\mathrm{fill}}^{2}}$$

in which *D*_particle_ and *n*_particle_ correspond to the diameter and refractive index of the nanoparticle, respectively, and *n*_fill_ is the refractive index of the filling matrix. One of the most commonly used matrices for this application is a cross-linked silicon-based elastomer, polydimethylsiloxane (PDMS). Depositing uniform three dimensional nanoparticle structures on hyrdophobic substrates has been one of the challenges to produce strongly iridescent materials. This was successfully achieved by using techniques such as dip-coating [16,29] or Langmuir-Blodgett deposition [30,31]. However, the commonly employed thin film deposition technique—spin-coating—is usually avoided, as it has a tendency to create non-uniform films due to the fast drying kinetics resulting from the quick rotation of the substrate [24]. Uniform nanoparticle photonic crystals from aqueous dispersions could be deposited on PDMS substrates by solving the wetting issue through a time-consuming chemical surface functionalization of the substrate [32].

Here, we introduce an innovative hybrid material based on spin-coated monodisperse polystyrene (PS) nanoparticles coated with a thin metallic layer and embedded in a PDMS matrix. By increasing the wettability of PDMS using a surface plasma treatment, we could form spin-coated, crack-free, close-packed, three-dimensional photonic structures on PDMS which, once coated with silver (Ag), exhibit strong iridescent colors. After depositing a second layer of PDMS on top of the Ag-coated photonic crystals, we produce a mechanically resistant material that can repeatedly be used as photonic chemical sensor for numerous organic solvents. Unlike previous studies on photonic sensors fabricated using self-assembled PS nanoparticles [16,28,33], we demonstrate that this approach is not limited to water and alcohols but can be applied to the detection and identification of hazardous solvents such as CF, CB, and THF, which can contaminate water and represent a real danger to human health and the environment. Using a set of five test organic solvents, we observe the dynamic reflectance peak shifts that result from variations in *d*_111_ as a result of the PDMS swelling. Furthermore, we verified that the prepared materials can be employed to detect small amounts of hazardous solvents mixed in water and, consequently, our study opens the path to low-cost photonic sensors for water contamination detection.

## 2. Materials and Methods

**Figure 1:**
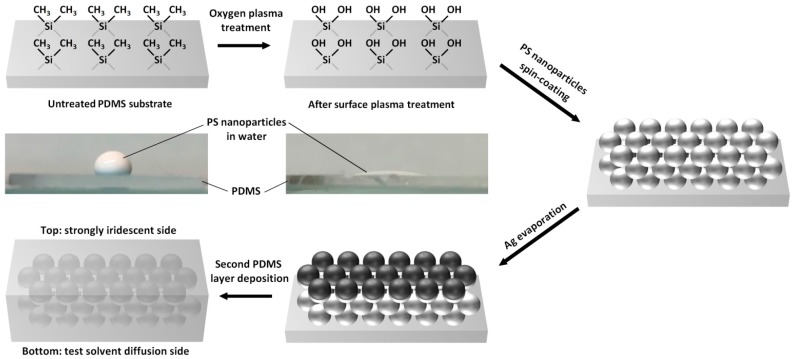
Schematic description of the multistep fabrication process for strongly iridescent hybrid photonic materials. The photographs correspond to PS nanoparticle aqueous dispersions deposited on PDMS substrates before and after oxygen plasma treatment.

A schematic representation of the fabrication procedure for the PDMS-based photonic sensors is presented in Figure 1. 2.5 × 2.5 cm^2^ PDMS substrates (Dow Corning, Sylgard^®^ 184, Midland, MI, USA) were fabricated by pouring a 10:1 mixture of the base and curing agent after intense mixing of the two components and depositing it in a square-shaped container before curing it at 80 °C for 2 h. The PDMS thickness (1.5 mm) was controlled by the deposited volume of uncured mixture. After the curing step, the PDMS substrates were exposed to oxygen plasma for 30 min using a pressure of 500 mTorr and a light intensity of 10 W at 10 MHz. The contact angle between the 600 nm diameter PS nanoparticle aqueous dispersion (Thermo Fisher 5060A, Waltham, MA, USA, size uniformity ≤ 3%, *n*_particle_ = 1.59 at 589 nm), and the PDMS substrates was accurately measured using a plug-in for the ImageJ 1.47v software, which follows a computational method that is described elsewhere [34]. The monodisperse PS nanoparticle dispersion diluted to 5 wt % was spin-coated on top of the modified surface PDMS substrates at 600 rpm for 1 min.

The PS-coated PDMS substrates used for metallization were placed in an evaporation chamber until a vacuum level of 10^−6^ Torr was reached and then an 70 nm-thick Ag layer was deposited on the substrates at an evaporation rate of 0.3 nm·s^−1^. The Ag-coated substrates employed for PDMS embedded photonic sensor fabrication were then once again placed in a square-shaped box and covered with the same volume of PDMS mixture that was cured in the same conditions as the substrate fabrication step. The resulting photonic sensors have a thickness of approximately 3 mm, which ensures that no buckling will occur when they are exposed to the organic solvents.

**Figure 2:**
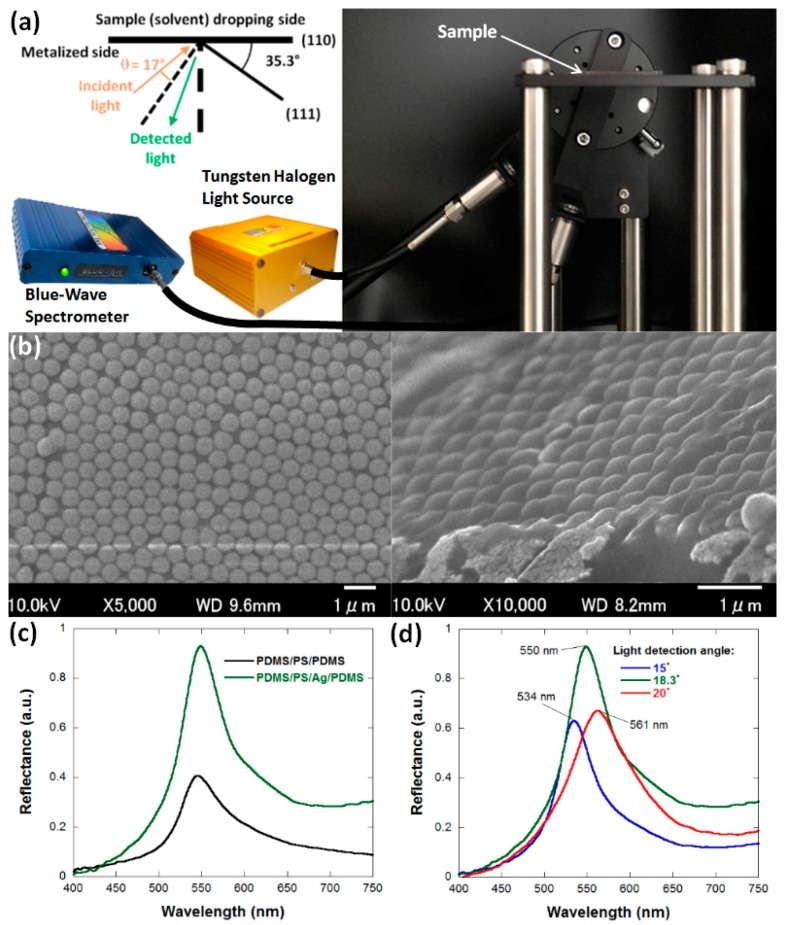
(**a**) Schematic representation and experimental set-up for the reflectance measurements; (**b**) top-view SEM image of the surface (left) and tilted-view SEM image of the cross-section (right) of Ag-coated PS nanoparticles deposited on plasma-treated PDMS; (**c**) reflectance spectrum of bare and Ag-coated PS nanoparticles embedded in PDMS for the third order of Bragg diffraction from the (111) plane observed at 17° with respect to the [111] direction; and (**d**) spatial and spectral distribution of the reflectance peak with incident light oriented 53.3° with respect to the vertical axis.

SEM images of the metalized PS nanoparticles that are deposited on PDMS substrates were collected using a Field Emission SEM (Hitachi, SU3500, Tokyo, Japan) at 10 kV and with magnifications of 5000× and 10,000×. The reflectance measurements were carried out by placing the samples on a horizontal stage with an incident light oriented 52.3° with respect to the vertical axis (Figure 2a). The light collected at an angle of 18.3° with respect to the vertical axis was analyzed using a BlueWave-VIS Spectrometer (StellarNet, Inc., Tampa, FL, USA) from 450 to 750 nm. These angles correspond to the Wulf-Bragg conditions (*θ* = 17°) for the close-packed nanoparticles in the (111) plane. The reflectance spectra were collected using a standard halogen lamp (StellarNet, Inc., Tampa, FL, USA, SL1 Tungsten Halogen Light Source) and normalized using the reflection spectra of Ag-covered glass substrates. All measurements were performed in the experimental room’s atmospheric conditions with temperatures of 25 °C and a relative humidity of 55%.

The PDMS swelling ratios were calculated by immersing the hybrid photonic materials (3 mm-thick, area of 2.5 × 2.5 cm^2^) into the various test solvents. The difference in weight before and after immersion for 1 h at room temperature and the molecular weight of the molecules was employed to calculate the amount of solvent that diffused into the hybrid material. Testing of the photonic materials as hazardous organic solvent sensors was performed using the same geometry as described above, and reflectance spectra were measured at regular time intervals after dropping 100 μL of each test solvent on the hybrid material surface (test solvent diffusion side in Figure 1). The sensitivity measurements were performed by gradually increasing the amount of THF placed on the photonic sensor surface. Similarly, 5 μL of THF were mixed into 95 μL of water, and the solvent mixture was deposited on top of the PDMS-based material to observe the changes in reflectance peak.

## 3. Results and Discussion

### 3.1. Strongly Iridescent Hybrid Photonic Films

After spin-coating the PS nanoparticles on a PDMS substrate, a 70 nm-thick Ag layer is deposited on top of these films, which are then covered with a second PDMS layer to ensure that the photonic structure is not damaged by repeated solvent diffusion. As demonstrated by the photographs in Figure 1, surface plasma treatment of the PDMS substrate is an essential step for ensuring that PS nanoparticles can be deposited from aqueous dispersions onto these high surface energy elastomeric substrates. Surface plasma treatment is a well-known technique that is often applied to improving the wetting properties of aqueous solutions onto hydrophobic layers such as poly(3,4-ethylenedioxythiophene) polystyrene sulfonate or PDMS [16,35,36].

The oxygen plasma treatment performed on PDMS modifies the functional groups present at its surface. In fact, the surface of pristine PDMS substrates is mainly composed of methyl groups. During plasma treatment, oxidation of these methyl groups takes place, which results in the formation of silanols that enable wetting from aqueous solutions and dispersions [37]. In the case of the PS nanoparticles dispersion we employed for our photonic material fabrication, average contact angles with the PDMS substrate of 107.5° and 24.9° were obtained before and after plasma treatment, respectively (Figure 1). This major change in contact angle and surface wetting properties enabled the spin-coating of wet layers of PS nanoparticle aqueous suspensions on PDMS substrates. Although the nanoparticles are dispersed in water, PS is intrinsically hydrophobic. The interactions between PS nanoparticles and the hydrophobic substrate (PDMS) are consequently likely to form uniform close-packed colloid photonic crystals. The scanning electron microscope (SEM) images in Figure 2b clearly demonstrate that a honeycomb close-packed arrangement of the PS nanoparticles on plasma-treated PDMS was successfully achieved. The photonic materials exhibits crack-free iridescence over areas as large as 5 cm^2^, confirming that, unlike previous attempts to fabricate PDMS-PS nanoparticle composites based on drop-casting, uniform arrangements were obtained [28]. Furthermore, coating the nanoparticles with a thin Ag layer considerably increased the reflectance of the PDMS-PS nanoparticle composites with values approximately 2.4 times higher than materials based on bare PS nanoparticles embedded in the silicon elastomer matrix (Figure 2c).

The face-centered cubic arrangements exhibit a (111) diffraction plane that is oriented at an angle of 35.3° with respect to the sample (Figure 2b). Consequently, for *θ* values, we consider the angle with respect to the [111] normal direction rather than the direction that is normal to the sample plane. To facilitate the visual detection of hazardous solvent traces, we selected the geometry in which *θ* has a value of 17°, as it results in a green structural coloration with a reflectance peak maximum around 550 nm (Figure 2c). Similarly to previous studies on photonic opal structures, we could observe a small spatial distribution of the diffraction peak, which may be a consequence of multiple diffractions from different Bragg planes that interact with each other (Figure 2d) [38]. This may also explain why the observed reflectance peak at 550 nm in Bragg conditions does not perfectly match the results from calculations using Equation (1), which predict a reflectance peak around 497 nm. The presence of packing defects (Figure 2b) and the dispersity in nanoparticle dimensions may also contribute to the observed differences between theoretical and experimental data. As the material present on the sample dropping side of these strongly iridescent multilayer films is PDMS, they may be employed to detect the presence of hazardous organic solvents, which can diffuse inside the silicon elastomer matrix [39].

### 3.2. Hazardous Solvent Detection in Biomimetic Photonic Sensors

To test the hybrid photonic materials we developed for hazardous organic solvent sensing applications, we selected five test solvents with various PDMS swelling ratios (Table 1). In addition to CF, CB, and THF, we used dichloromethane (DCM) and dimethoxyethane (DME), which are suspected of causing cancer or may damage fertility and unborn children.

**Figure 3:**
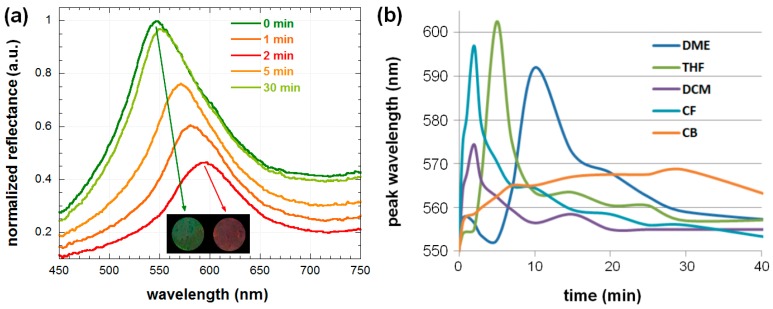
(**a**) Time-resolved reflectance spectra of 100 μL CF dropped from the non-metal side onto the hybrid photonic sensor; (**b**) time-dependent evolution of the reflectance peak wavelength of various solvents dropped on the hybrid photonic sensor. The inset in (**a**) corresponds to the observed sample with an area of 5 cm^2^ before deposition of CF and 2 min after the deposition.

The five tested solvents exhibit dynamic shifts of the reflectance peak that were initially observed at 550 nm. Taking into account Equations (1) and (3), the measured red shifts (Figure 3) of the reflectance peak either originate from an increase in the distance between neighboring centers in the (111) plane or larger *n*_eff_ values. The refractive indices of the test solvents (*n*_solvent_) are summarized in Table 1. Interestingly, although THF and PDMS are relatively similar from an optical point-of-view, with *n*_THF_ and *n*_PDMS_ having values of 1.40 and 1.41, respectively, a 50 nm red shift of the reflectance peak can be observed, which suggests that the key parameter for the response from the photonic sensor is the PDMS swelling ratio. In fact, CF, which has a refractive index of 1.45 but almost the same swelling ratio as THF, yields similar values of reflectance peak shift.

Using the solvent with a high swelling ratio (CF), we verified that the changes in reflected color from green to red (597 nm) can be visually recognized within a few minutes (inset of Figure 3a). In fact, the five test solvents can be categorized in three classes of swelling ratios with values close to 18 (CF and THF), 12 (DME), and 8 (DCM and CB) mmol/g of PDMS, respectively. These three classes of solvents yield shifts of approximately 50 nm, 40 nm, and 20 nm, respectively. Consequently, it is safe to assume that the working mechanism of these biomimetic photonic sensors is very similar to the color-tuning properties of the chameleon skin and principally relies on a deformation of the photonic crystal lattice upon swelling of PDMS by the solvents (increase in *d*_111_) [10]. Note that the standard deviation for 10 measurements using the same solvent is less than 1 nm, which implies that even small differences in reflectance peak wavelength (e.g., 601 and 597 nm for THF and CF, respectively) can be employed to identify the nature of the solvent that diffuses into the photonic sensor. Nonetheless, to ensure that each solvent can be precisely identified, we used a dynamic approach based on the solvent diffusion rate into the PDMS matrix. As displayed in Figure 3b, CF and DCM reach their maximum reflectance shifts within 2 min, whereas THF, DME, and CB take longer times of approximately 5, 10, and 30 min, respectively.

**Figure 4:**
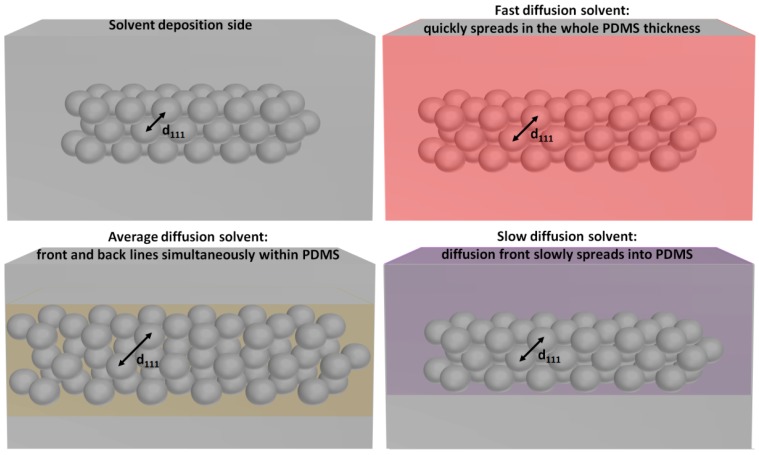
Schematic representation of the increased distance between adjacent PS nanoparticles embedded in the PDMS matrix when solvents with fast, average, and slow diffusion speeds are deposited on the surface of the photonic sensors.

The time-dependent measurements not only allow for a better recognition of the solvents but also provide important information for understanding the differences observed in reflectance shifts from solvents with similar PDMS swelling ratios. For instance, CF and THF have PDMS swelling ratios of 17.7 and 17.8 mmol/g, respectively. Nonetheless, as CF has a higher refractive index compared to THF, a larger red shift is expected. Repeated measurements confirm that the reflectance measured from the sensors in the presence of THF is red-shifted by approximately 5 nm as compared to the results from CF. Our hypothesis for this contradictive result is that the solvent diffusion kinetics, which determine the amount of solvent present in the vicinity of the PS nanoparticles at a given time, will affect the increase in *d*_111_ as represented in Figure 4.

As the reflectance peak shifts back to the initial position (550 nm) after a certain time, we neglected the effect of PS solubility on the various test solvents and considered that the solvents preferentially diffuse into PDMS. When CF is deposited on top of the photonic sensor, it quickly diffuses inside PDMS, which results in an almost homogenous distribution of the solvent molecules inside the whole elastomeric matrix thickness (approximately 3 mm). On the other hand, in the case of THF, the diffusion is slower and, consequently, the solvent swollen area gradually moves in the vertical direction within PDMS. The schematic representations in Figure 4 are exaggerated for clarity of presentation. In the case of fast diffusing solvents (e.g., CF), a smaller amount of solvent molecules remain in the direct proximity of the PS nanoparticles at the time when the maximum reflectance peak shift is recorded. This also explains why a smaller shift is observed for CF with respect to THF, which has slower diffusion kinetics even though the two solvents have similar swelling ratios (measured by immersing the photonic sensors into the various solvents). A slower diffusion rate consequently seems to be beneficial for precise measurement of the maximum peak shift due to swelling of the PDMS matrix. Nonetheless, if the diffusion of the solvent inside PDMS is too slow, the distance between the diffusion front and back will increase, which will also result in less solvent molecules in the vicinity of the PS nanoparticles at a given time. Consequently, a larger red-shift can be observed when comparing DCM to CB despite the fact that they have similar swelling ratios. The hypothesis of time-dependent swelling of PDMS suggests that the reflectance shifts should also depend on the volume of solvent that is in contact with the sensor.

We verified the detection threshold (sensitivity) of the photonic sensors we developed here by varying the amounts of solvents used as test samples (Table 2). All the reflectance wavelengths in Table 2 correspond to the maximum shift observed during each test. The time at which these maximum shifts were observed varies depending on the test solvent. Additionally, considering the experimental error on the reflectance peak measurements, we can suppose that the minimum volume of solvent that can be detected by the photonic sensor is 5 μL (shifts larger than 5 nm). To verify whether this detection threshold can be applied to, for example, THF mixed in water, we prepared a low concentration (5 vol %) mixture of THF in water. The mixed solvent was deposited on the photonic sensors, which resulted in a reflectance peak wavelength of 569 nm collected 5 min after deposition. As this value is very close to the one measured for 5 μL of THF deposited on the sensor, we can speculate that all the THF molecules mixed in water diffuse from the mixed solvent into the PDMS based photonic sensors. These sensors consequently have a great potential as innovative technology for precisely and rapidly detecting the presence of hazardous, solvent-contaminated water.

## 4. Conclusions

In summary, we have successfully fabricated strongly iridescent hybrid photonic sensors based on self-assembled spin-coated PS nanoparticles on plasma-treated PDMS substrates. The addition of the reflective Ag layer resulted in strong structural coloration properties that can be easily observed by naked eye. The formation of close-packed structures was confirmed by SEM measurements, and we employed the green Bragg reflection peak (*λ* = 550 nm) to probe the diffusion of hazardous organic solvents into the photonic sensor.

Using a set of five organic solvents, we verified that these multilayer nanostructured materials can be used for the detection and identification of hazardous water contaminants. Direct visualization of the changes in reflectance peak wavelengths for a given amount of solvent can be highly beneficial for rapid analysis of the water purity with respect to these organic solvents, which can be harmful to the human health and the environment. After closely studying the behavior of the various solvents and correlating the observed reflectance peak shifts with the parameters present in the Bragg diffraction equation, we concluded that, similarly to the chameleon skin, the changes in visible color of the sensors strongly depend on the PDMS swelling by the test solvent. As each solvent has a different diffusion rate into PDMS, reliable contaminant identification can be achieved by analyzing the data in terms of time-dependent reflectance peak shift.

Lastly, we verified that hazardous solvent amounts as low as 5 μL can be detected using the hybrid photonic sensors, even when they are tested as low concentration contaminants in water. This opens the path for facile and rapid recognition of a variety of hazardous solvents that can either affect some organs such as the liver or the lungs but also result in infertility or increase the probability of harm to unborn children. As this technology can be easily employed as simple water purity test in developing countries in which sometimes no strict rules on industrial solvent disposal exist, the sensors we produced for our study could find major applications in global safety measure implementation.

## Figures and Tables

**Table 1:** Summarized data on tested solvent and observed reflectance peak shift when 100μL of solvent are deposited on the hybrid sensor.

Solvent	THF	DME	CF	CB	DCM
Refractive index (*n*_solvent_)	1.40	1.38	1.45	1.52	1.42
PDMS swelling ratio (mmol/g of PDMS)	17.8	11.6	17.7	7.7	7.9
Reflectance peak (nm)	601	592	597	569	575

**Table 2:** Photonic sensor reflectance peak wavelength with respect to deposited amount of solvent.

**Solvent Amount (μL)**		0	2	5	10	20	50	100
**Reflectance Peak (nm)**	**THF**	550	552	567	574	599	602	601
	**DME**	550	555	562	571	578	591	592
	**CF**	550	551	558	569	589	599	597
	**CB**	550	552	557	567	571	570	569
	**DCM**	550	554	561	569	574	574	575
